# Supplementary material for: Shrinking to bird size with dinosaur-level cancer defences: Evolution of cancer suppression over macroevolutionary time
Source: PLoS Comput Biol. 2025 Sep 9;21(9):e1013432. doi: 10.1371/journal.pcbi.1013432 (PMC12435733; doi:10.1371/journal.pcbi.1013432)
Supplement: S1 Text — (PDF) [file pcbi.1013432.s001.pdf]

S1 Text
Shrinking to bird size with dinosaur-level cancer defences: evolution of cancer suppression over macroevolutionary time

E. Yagmur Erten[a,b,c,d]*, Marc Tollis[b,e], Hanna Kokko[a,f,g]

[a] Department of Evolutionary Biology and Environmental Studies, University of Zurich, Zurich, Switzerland.
[b] Arizona Cancer Evolution Center, Arizona State University, Tempe, Arizona, United States.
[c] Department of Biological and Environmental Science, University of Jyväskylä, Jyväskylä, Finland.
[d] Groningen Institute for Evolutionary Life Sciences, University of Groningen, Groningen, The Netherlands.
[e] School of Informatics, Computing, and Cyber Systems, Northern Arizona University, Flagstaff, Arizona, United States.
[f] Institute of Organismic and Molecular Evolution, University of Mainz, Mainz, Germany.
[g] Institute for Quantitative and Computational Biosciences, University of Mainz, Mainz, Germany.
*Corresponding author: e.y.erten@rug.nl

Here we test the robustness of our results to different parameter values and modelling assumptions for: the area size used to estimate the effective population size (Fig A), the distribution of mutation steps (Fig B), the number of time bins (Fig C), presence of allometric relationships between body size and i) extrinsic mortality, ii) population size (Fig D), and the implementation of flight (Fig E). For simplicity, in these robustness tests, we will focus on three lineages: constant-sized large flightless lineage, constant-sized small flightless lineage, and miniaturised flightless lineage. For completeness, we also expand the figure 2 and 3 in the main text to include calculations for the constant-sized lineages (Fig F-G).

Area size used to estimate the effective population size

In our model, population density scaled with body size and in the main text we used an area of 10^4 to calculate the effective population size as $N_\mathrm{e} = 10^{4-0.49(\log_{10} M+3)+1.96}$. Here we tested the effect of using different area sizes: 10^3 (with $N_\mathrm{e} = 10^{3-0.49(\log_{10} M+3)+1.96}$) and 10^5 ($N_\mathrm{e} = 10^{5-0.49(\log_{10} M+3)+1.96}$).

Using different area sizes changed the range of mutation rates that led to evolutionary lags in fitness (i.e. the positioning of the mutational boundary), but did not otherwise alter our results qualitatively as the evolutionary lags persisted for both area sizes (Fig A). Location of the mutational boundary was higher (i.e. at higher mutation rates) for a smaller area (10^3) and lower (i.e. at lower mutation rates) for a larger area (10^5), compared to the results in the main text (for an area of 10^4). This is in line with the expectation

for an increased strength of selection in large populations (Lanfear et al., 2014), which in our model results in an increased chance of substitutions to reduce cancer defences, and therefore to reduce fitness lags, in the miniaturised lineage.

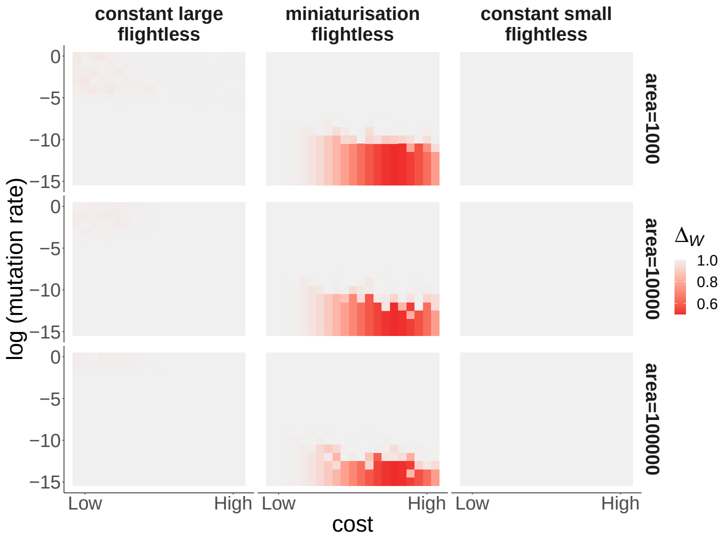

Figure A: Evolutionary lags in fitness for different area sizes. Each panel shows the cost of cancer defences (x axis: $\log_{10} c$ ranging between -4.1 and -0.1) and mutation rate (y axis: $\log_{10} m$ ranging between -15 and 0). Columns: different scenarios; rows: different area sizes, as indicated in the figure. Middle row shows the area used in the main text. The scale for evolutionary lag in fitness, Δ_W, is shown in the legend. Positioning of the mutational boundary (here inferred from the occurrence of evolutionary lags in fitness) differed, but the results were otherwise qualitatively similar across different area sizes. Results are reported for the number of oncogenic steps n=3 and the rate of oncogenesis k=0.0001 for all scenarios.

Distribution of mutation steps

In our model, we assumed that the distribution of mutational steps reflects the observation that advantageous mutations are rarer than deleterious mutations (Eyre-Walker and Keightley, 2007). For our model this translated into decays in cancer defences being more likely compared to gains. Furthermore, beneficial mutations with large effect were unlikely to happen. For this, we used a generalized extreme value random distribution with parameters (k=–0.75, σ=1, μ=–1), bound between (-1,1). To test the robustness of our results to the distribution of δ, we re-ran our macroevolutionary simulations with mutation steps following a Gaussian distribution with $\delta \sim N(0, 0.3)$, also bound between (-1,1). We observed no qualitative difference in evolutionary lags between the two mutational distributions (Fig B).

Number of time bins

In the main text, we divided macroevolutionary time T into 4000 bins of 60000 years each. In these bins we kept body size and the traits that scale with body size (e.g. effective population size) constant. Changing the number of time bins to a lower (1000) or a higher (16000) value did not affect our results qualitatively (Fig C).

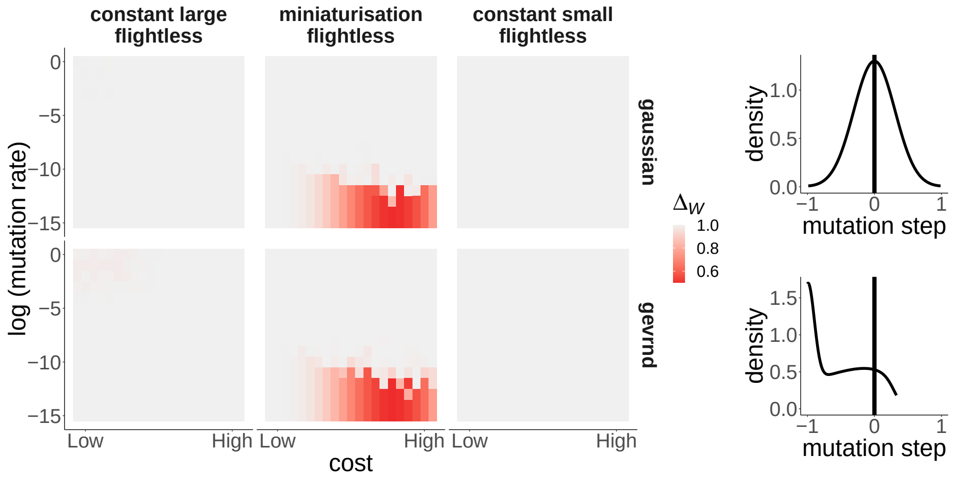

Figure B: Evolutionary lags in fitness for different mutation step distributions. Panels, columns, and the figure legend as in Fig A; rows depict two different mutation distributions: Gaussian (top) and a generalized extreme value (bottom; used in the main text). The evolutionary lags in fitness were similar between the two distributions. Parameters as reported in (Fig A), for an area of 10^4.

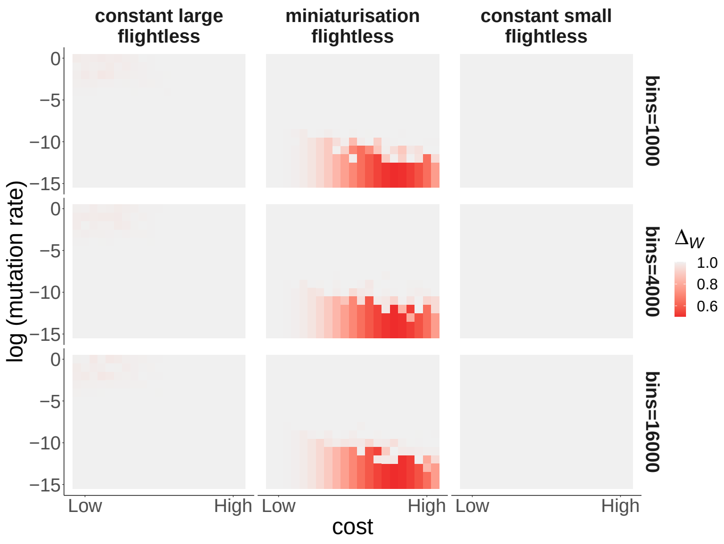

Figure C: Fitness lags for different numbers of time bins. Panels, columns, and the figure legend as in Fig A; rows: the number of time bins. The middle row shows the value used in the main text. Fitness lags and positioning of the mutational boundary were similar across different numbers of time bins. Parameters as reported in (Fig A), for an area of 10^4.

Allometric relationships between body size, extrinsic mortality, and population size

In our model, we assumed that the effective population size and extrinsic mortality scaled with body size. Here, we tested the robustness of our results to the existence of these scaling relationships by looking at fitness lags when i) population size was constant (and extrinsic mortality scaled with body size), and ii) extrinsic mortality was constant (and population size scaled with body size). Removing the allometric relationship between the body size and population size resulted in a smaller effective population size in the shrinking lineage compared to the baseline model. As a result, the mutational boundary was slightly higher (Fig D), in line with the results shown in Fig A. Keeping the extrinsic mortality at a constant level, equal to the dinosaurian ancestors, lowered the fitness lags

below the mutational boundary. Having lower extrinsic mortality and longer lifespan than in the baseline scenario allowed the lineage with constant extrinsic mortality to better take advantage of the ancestral defences, similarly to the flighted miniaturised lineage that also had an extrinsic mortality reduction (Fig F).

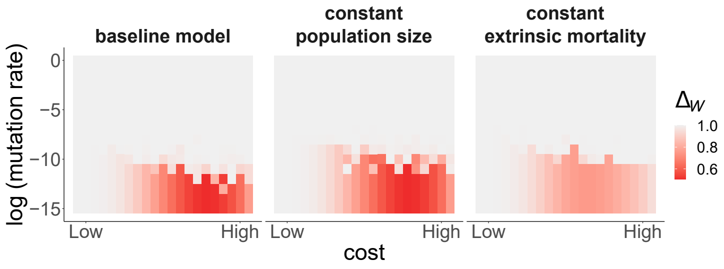

Figure D: Fitness lags when allometric relationships are switched off. All lineages are miniaturised and flightless. Columns depict the evolutionary fitness lags for, left-to-right: i) baseline model, ii) the model where the allometric relationship between body size and population size is switched off, and iii) the model where the allometric relationship between body size and extrinsic mortality is switched off. The evolutionary lags persisted and the results were in line with Fig A for the scenario (ii) and Fig F for the scenario (iii). Parameters as reported in (Fig A), for an area of 10^4.

Timing of and extrinsic mortality reduction by flight

In the main text, we reported our results with flight innovation occurring at 170 Mya and reducing extrinsic mortality with the coefficient r^{-1}=3. Here we show the results for different timing (180 Mya and 160 Mya) and extrinsic mortality reduction (r^{-1}=2 and r^{-1}=4) values (Fig E). Different flight time scenarios resulted in similar evolutionary lags. In line with our results in the main text, evolutionary lags in fitness shrank with an increase in r^{-1}: reductions in extrinsic mortality rate lengthen the lifespan and allow the 'lagged' lineages to benefit more from their ancestral defences.

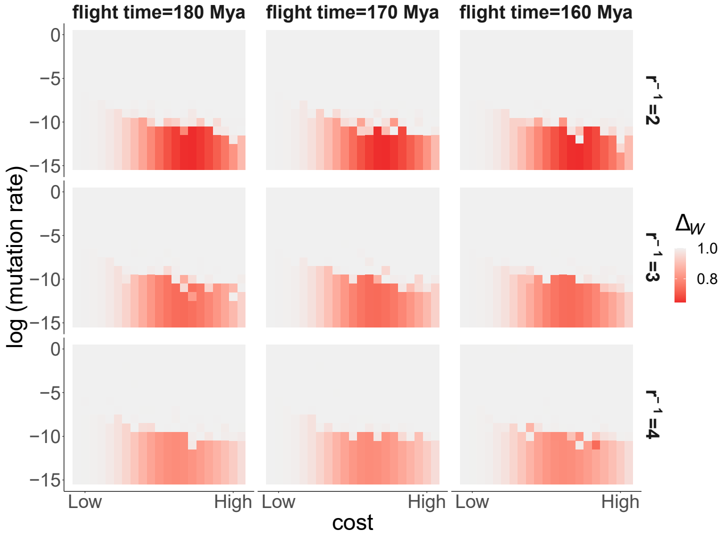

Figure E: Fitness lags for different flight scenarios. Rows: different extrinsic mortality reduction coefficients (r^{-1}; columns: different time points for the flight innovation; panels and figure legend as in Fig A. Middle row and middle column (flight time=170 Mya, r^{-1}=3) is the parameter combination reported in the main text. The fitness lags became smaller as r^{-1} increased, while the timing of flight acquisition did not affect the results qualitatively. Parameters as reported in (Fig A), for an area of 10^4.

Comparisons with the constant-sized lineages

To complement our results in the main text, here we present the results for the constant-sized lineages in addition to the miniaturised ones. As the constant-sized lineages' optimal cancer defences did not change through time (as opposed to the miniaturised lineages that had a shift in their optimal value), their cancer suppression level remained at a mutation selection-balance above the mutational boundary and at their optimal level below the mutational boundary (Fig Fa). Consequently, as opposed to the miniaturised lineages, they did not experience any lags in lifespan or fitness below the mutational boundary (Fig Fb-c).

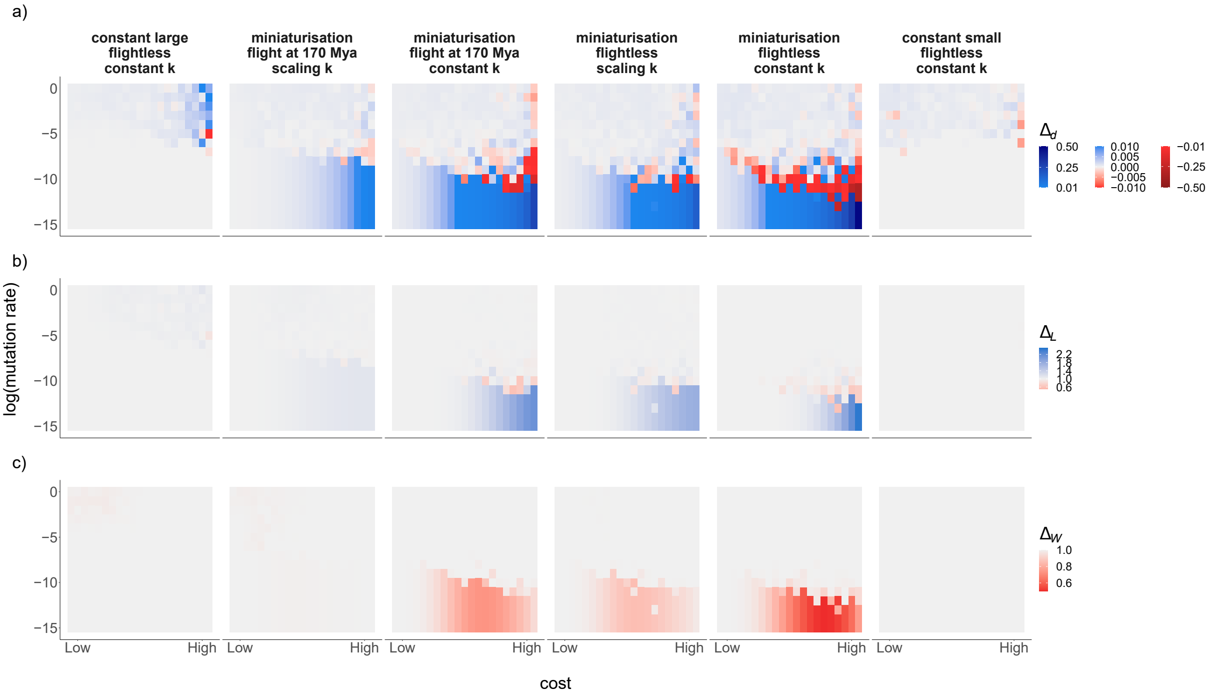

Figure F: Evolutionary lags for all scenarios. Columns depict different scenarios as indicated, rows show evolutionary lags in (a) cancer defences (Δ_d), (b) lifespan (Δ_L), and (c) fitness (Δ_W). Panels as in Fig A. We observe no evolutionary lags in fitness in the constant-sized lineages. Parameters as reported in (Fig A), for an area of 10^4.

Cancer defence level and the probability of dying from cancer was overall higher, and lifespan was longer in the dinosaur-like lineage (constant large, flightless, constant k in Fig G), compared to the mammal-like one (constant small, flightless, constant k in Fig G). When at an evolutionary lag, miniaturised lineages with constant k had the lowest probability of dying from cancer, even when compared to the mammal-like one (Fig Fb). Finally, flightedness was the main determinant of lifespan in miniaturised lineages (Fig Gc), with flighted ones having lifespans comparable to their dinosaurian ancestors, and flightless ones having short lifespans like the mammal-like comparison.

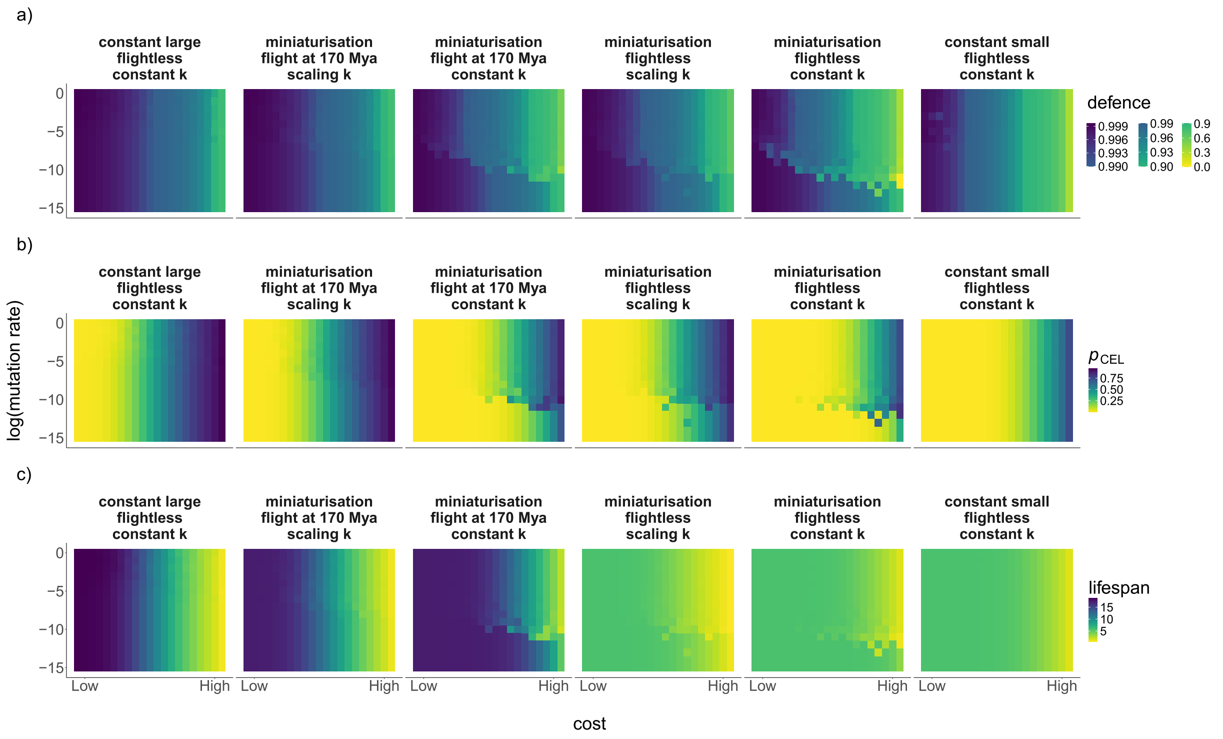

Figure G: Cancer defence and mortality, and lifespan for all scenarios. Columns depict different scenarios as indicated, rows show (a) cancer defence level, (b) probability of dying from cancer (p_{CEL}), and (c) lifespan (in years). Panels as in Fig A. Parameters as reported in (Fig A), for an area of 10^4.

References

Eyre-Walker, A. and Keightley, P. D. (2007). The distribution of fitness effects of new mutations, *Nature Reviews Genetics* **8**(8): 610–618. doi:10.1038/nrg2146

Lanfear, R., Kokko, H. and Eyre-Walker, A. (2014). Population size and the rate of evolution, *Trends in Ecology & Evolution* **29**(1): 33–41. doi:10.1016/j.tree.2013.09.009
