# Supplementary material for: Shrinking to bird size with dinosaur-level cancer defences: Evolution of cancer suppression over macroevolutionary time
Source: PLoS Comput Biol. 2025 Sep 9;21(9):e1013432. doi: 10.1371/journal.pcbi.1013432 (PMC12435733; doi:10.1371/journal.pcbi.1013432)
Supplement: Attachment — Submitted filename: Response to reviewers - 2025 01 20 Final.docx [file pcbi.1013432.s003.docx]

21 01 2025

Dear Editor,

We hereby submit a revised version of our manuscript PCOMPBIOL-D-24-01081 **“Shrinking to bird size with dinosaur-level cancer defences: evolution of cancer suppression over macroevolutionary time”** for review in *PLOS Computational Biology*.

Please find below our response to the reviewer comments.

We hope you find our paper suitable for publication in *PLOS Computational Biology*.

Best regards (on behalf of the authors),

Dr. E. Yagmur Erten

Groningen Institute for Evolutionary Life Sciences (GELIFES)

University of Groningen

The Netherlands

Response to Reviewer Comments

Below we address the reviewer comments and describe how we revised our manuscript. Reviewer comments are shown in black and our responses are in blue. The line numbers correspond to those of the version with track changes.

**Reviewer #1**

This work models multiple possible scenarios for the evolution of cancer defences in birds after divergence from their dinosaur ancestors.

A series of macroevolutionary simulations are run to recreate multiple possible scenarios to investigate the potential impacts of body size reduction and the gain of flight capability may have on evolution of cancer defences in birds.

Many of the parameters used are carefully defined and justified by the authors based on the literature and evolutionary principles.

We thank the reviewer for these nice statements and also for their critical comments below.

However, there was little information provided regarding the basis on which the range of values for m (mutation rate per genome per generation) were selected. This is important because the authors show there is a clear ‘mutation rate boundary’ effect that leads to dramatically different outcomes depending on whether rates of mutations that impact cancer defences are high or low.

Likewise, it is unclear how many loci impact cancer defences in these simulations, and what their combined length is. While anti-cancer mechanisms are complex and the total number of possible loci controlling them is likely not known at this point, the number of length of canonical tumour suppressor genes would serve as a useful baseline, given the strong conservation of their sequence and function across vertebrates.

We selected a broad range (15 orders of magnitude!) of mutation rates, following the justification below.

1) For single mutations: According to Bergeron et al. 2023, mutation rate per site per generation is 1.01 × 10^−8^ in birds, with 95% CI of the mean between 6.10 × 10^−9^ and 1.42 × 10^−8^. Bird genome sizes range between ∼0.96–2.2 Gb (Kapusta et al. 2017). For mutation rate per genome per generation, the lowest value would be 5.83 and highest value would be 31.24, taking these ranges into account.
2) However, mutation rate varies across the genome (as well as within species; Bergeron et al 2023)
3) There is no good estimate available for the proportion of sites that affect cancer defences.
4) For indels, the frequency is still lower than single mutations.

We model the mutation effects as a distribution of fitness effects, skewed towards more deleterious than beneficial mutations, in line with the literature (Eyre-Walker 2007). It is not straightforward to estimate how many loci can impact cancer defences in one organism, let alone across macroevolutionary time in organisms with different body size or genome size. In this situation, we considered it wise to err on the side of considering the entire range from very small to very large rates, with the former likely to be an underestimate and the latter an overestimate of what happens/happened in reality. Our thinking is that if future estimates can yield precision of where to look in our figures, we have already covered the realistic area.

Note that the distribution of effects we model can implicitly account for different scenarios, e.g. one locus of large effect (e.g. when the mutation effect is large and deleterious) or several loci of small effect (e.g. many small incremental changes).

The simulations yielded intriguing and insightful findings that provide understanding of the evolutionary tradeoffs between cancer defence and other advantageous traits in birds.

However, overall the paper is somewhat inconclusive in regards to what is the most likely scenario of evolution of cancer defences in birds. Other than ruling out the loss and subsequent regain of cancer defences as not being parsimonious, it’s hard to tell which evolutionary scenario modelled here is most likely.

We agree with this assessment by the reviewer. The inconclusivity arises from the fact that we aimed to produce a modelling study – not a genomic one (had we done the latter, we probably would have aimed for a more genomic data-oriented journal in the PLOS family).

The manuscript would be improved by data that could support and solidify its main messages.

Numerous bird genomes are now available, covering a range of lineages. These data could be used for comparative analysis among birds and against other vertebrates classes, particularly in the aforementioned canonical tumour suppressor genes, to determine whether mutation rates correspond to capability for flight and body size.

Beyond that, data on lifespan, brood size and cancer incidence could be compared within both flighted and flightless bird lineages that show large variation in body size, to assess validity of other associations predicted by the authors’ models.

We thank the reviewer for these suggestions. We agree that a comparative genomics analysis across birds, comparing birds with other vertebrates, and performing comparative analyses of life history data are all extremely worthy goals. Given the scale of such an endeavour, we must consider them beyond the scope of our current study, however. Here, our goal is to introduce an innovative macroevolutionary model: see Fig. 1 for how many concurrent processes are considered; we show other researchers how this can be done, and how the precise outcomes can be quantified. We do not wish to downplay the importance of future work; indeed we now write: “With the growing availability of genomic, demographic, and oncological data across species, comparative analyses between birds and against other vertebrates are now very well-positioned to uncover the mechanisms underlying avian cancer robustness.” (lines 606-609)

**Reviewer #2**

This manuscript by Erten et al. explores the evolutionary dynamics of cancer suppression in birds, proposing that birds may have retained ancestral cancer defenses from their large-bodied, dinosaurian ancestors. The study models how innovations like flight and metabolic scaling affect cancer risk and longevity, suggesting that these adaptations helped maintain or re-activate high cancer suppression in birds, despite miniaturization. The findings provide insights into the relationship between evolutionary history, life history traits, and cancer resistance in birds. The manuscript is well written, logical and I only have a few, rather minor comments.

We are really glad to hear that the MS is well written and thank the reviewer for their comments.

Line 5 (also abstract): While it was long believed that cell division is one of the key sources of mutations, it seems that most-mitotic cells also accumulate mutations at pretty much similar rates as dividing ones. Read this: <https://www.nature.com/articles/s41586-021-03477-4>

We thank the reviewer for this reference. We revised this accordingly (abstract and lines 5-8).

Line 13: a priori should be italicized

Thank you, we fixed this (line 17).

Line 31: birds experience not just a delayed onset of senescence, but also a slower rate of senescence: see e.g. https://royalsocietypublishing.org/doi/full/10.1098/rstb.2019.0744

We thank the reviewer for this reference, which is useful indeed, though to be precise, the paper quantifies reproductive senescence, which is a somewhat different topic – though reproductive and actuarial senescence are correlated. We also added an older reference on actuarial senescence, see lines 38-39.

Line34: "experience lowered extrinsic mortality from predation" - not just predation. Flying and migration over long distances serves to avoid food shortages, so also , a pattern repeated in 34 flying mammals [32,34,37] which, too, live long for their body size.

There is actually some discrepancy in the literature when it comes to migration and its effects on longevity. Soriano-Redondo et al. 2020 state that migration (after correcting for various confounds) associates with shorter lifespans in birds and mammals, while Winger & Pegan 2021 report higher adult survival with increasing migration distance in passerines. It is difficult to say for sure what accounts for these differences, but note that it is possible that migration allows a species to colonize breeding grounds where it would not be able to persist year-round at all, and the comparison to a non-migrant may not be of an “all else is equal” nature. Thus, the ability to develop (long-distance) migration as a result of having wings may or may not have improved lifespans on average. Given the complexities of migration, we decided against making the story more complex by discussing it, as our argument also works without it and is more parsimonious that way.

Line 497-500: long and difficult to follow. e.g. " incorporated burrow-living adaptations into their cancer defence" - burrow-living is not a cancer defence I believe, or at least it's not the primary reason. Also " losing the defence would be selected against" could be said as "maintinting the defence would be selected for" to simplify the text.

We thank the reviewer, we revised this sentence for increased clarity (lines 578-583).

Line 501.504: an example could help to understand what you refer here.

Cancer suppression in birds is not as well-studied as in mammals, which makes this task difficult. The explicit studies that exist tend to have a medical focus with an understandable emphasis on similarities to humans (e.g. Rietveld et al. 2001, Niebora et al. 2024), and this forces studies on birds to focus on cancer-related genes that are known to play this role in humans (e.g. Meitern et al. 2020). This is unlikely to be the whole story, however. Mammalian taxa, whenever studied, show strong differences, as illustrated by e.g. elephants and whales: the former have a higher copy number of p53 (Abegglen et al. 2015) while the latter rely, loosely speaking, on ‘more careful’ DNA replication to begin with (Tollis et al. 2019, see also Firsanov et al. on BiorXiv, doi.org/10.1101/2023.05.07.539748). Since the bird-mammal divergence is very much older than that between whales and elephants, we do not doubt the existence of adaptations that are unique to birds, but there is a severe lack of information on these (see, however, evidence that avian fibroblasts are more stress-resistant than those of mammals, Harper et al. 2011).

Since we first submitted our MS, a new study (Kapsetaki et al. 2024) has become available, that documents cancer rates across birds; while useful in other places in our MS (we have now added it in several places), it remains rather uninformative of precisely worked out examples.

We now give a brief summary of this state of matters on lines 490-501 and have a speculative example about how bird cancer suppression could relate to other avian traits on lines 586-591.

The manuscript argues that metabolic rate in birds increased compared to their dinosaur ancestors. Are the authors sure of this? I believe metabolic rate closely correlates with body mass across all vertebrates. do the authors maybe mean mass-specfific metabolic rate? Which is indeed higher in birds than in size matched mammals/reptiles, etc. If so, all text and figures need to be correled accordingly.

We thank the reviewer for bringing this to our attention. Indeed, we mean mass-specific metabolic rate. We have revised our phrasing throughout the manuscript accordingly.

**Reviewer #3**

The authors ask whether the reduced frequency of cancer in existing birds may be a heritage of dinosaurs, which may have had higher defences against cancer due to their large size. To address this possibility, they develop a mathematical model to produce macroevolutionary simulations. The hypothesis being correct is not trivial. Higher cancer defences come with reproductive costs, which will be typically counter selected. Using the model, the authors identify scenarios where this is actually possible: if costs are minimal (obviously); if birds undergo additional changes (here, metabolic) that increase the chances of developing cancer; and if they acquire additional properties (here, the ability to fly) that may decrease the extrinsic death rate and allow a longer life-span.

The paper is interesting. Although the authors took commendable efforts to identify reasonable parameter ranges, the results are very speculative. Yet, I believe this is appropriate for PLoS Computational Biology. The manuscript has the merit to discuss an intriguing idea, and thus I support it for publication. Yet, I think it can be improved, especially in terms of data representation.

We thank the reviewer and agree: the way that a computational study can explore a very wide range of parameters was indeed the reason we chose this journal as our outlet (pending acceptance of course).

Major comments

1 - The model has a large number of parameters. The authors decided to investigate the model changing two of them: mutation rate and cost of cancer defence. What is the rationale for choosing these two?

First, a small remark: while we varied mutation rate and cost of cancer suppression in a more fine-grained way than the other parameters, we did look at the robustness of our results to different parameter values and modelling assumptions (Supplementary Information), and changed extrinsic mortality reduction by flight (Sup. Figure S5), area (Sup. Figure S1), and distribution of mutation effects (Sup, Figure S2).

Then, the answer to why our main results focus on mutation rate and cost of cancer suppression. These two parameters are the most relevant ones for our question; mutation rate directly affects how fast cancer defences can evolve (a lag would not exist and persist over macroevolutionary time scales if mutations were abundantly available), and the cost determines the strength of the life history trade-off between cancer suppression and reproductive success. The cost of cancer suppression is a much discussed phenomenon in the literature at present and a new study by Kapsetaki et al. 2024 (which we already mentioned above) strengthens the case that cost is a key parameter, as it documents a positive relationship between clutch size and malignancy prevalence across 56 bird species. We weave this rationale into our manuscript (lines 495-497, 533-542, and 543-554).

2- After reading the paper, the answer to the question very clearly stated in the title is not super clear, at least to me. One is left with the impression that almost everything is possible. A few suggestions/questions:

We have edited the title mildly to avoid any feeling that we might be overselling our study.

- is there any evidence that mutation rate and cost can vary in the range they use for the density plots? Which of the many scenarios is closer to available data? And so, which area of the density plots is more realistic?

We understand the point raised by the reviewer concerning the realistic values of mutation rate and cost.

Mutation rate: See our response to Reviewer 1.

Cost: There is evidence for the relationship between cancer and reproduction; for instance a classical example from the literature is *Xiphophorus* where males that have the Xmrk genotype have larger body size (which is sexually selected) but are also more likely to develop melanoma later in life (Fernandez & Bowser, 2010). As mentioned above, there is now new data regarding the cost in birds, where clutch size covaries positively with malignancies (Suppl. Figure 16A in Kapsetaki et al. 2024). It would be somewhat dangerous, however, to attempt to estimate the strength of this effect from correlational data only (Dujon et al. 2023 provide other examples), as each species has evolved to a different point along a risk-benefit curve, and what is an “acceptable” risk (from a life history optimality point of view) also depends on lifespan. We decided to look at a broad range of values from very low to very high cost of cancer suppression precisely because of the difficulties to quantify the relationship between cancer suppression and reproductive success. In this sense, our response is similar to that of the mutation rate.

We now cite the papers above in appropriate places in the MS.

- the abstract could be more direct making explicit the 'under certain assumptions' cited there.

We have now made this part more explicit in the abstract: “If the evolution of cancer suppression is mutation limited, due to e.g. pleiotropy, birds may have also kept excessive dinosaurian cancer defences, possibly explaining their low cancer risk.”

3- One general point: changes in metabolism may increase the chances of developing cancer, and thus, the authors argue, would avoid eroding cancer defences in shrinking lineages. In this case, would one expect a different or similar tendency to develop cancer in birds and in mammals? More in general, if miniaturisation, flight, scaling k represents existing birds and constant small, flightless constant k is mammal-like, in which parameter range do the first show less predisposition for cancer? From Figure S6 one would not notice big differences for DL and DW. Figure 4B and 2d also do not show big changes between these two conditions. I think this point (comparison mammal-like vs birds in terms of cancer propensity in the model) should be better discussed, since the original observation is precisely the difference in cancer propensity between birds and equally sized mammals.

If bird mass-specific metabolic rate is higher than the mammal one, and if mass-specific metabolic rate correlates with the rate of oncogenesis, all else being the same, we would expect a bird to have higher cancer risk compared to a mammal of same size. We included metabolic rate scaling as a scenario in our study for completeness, as it is often included in allometric scaling studies and has already been discussed in the context of cancer (e.g. Nunney 2020). However, as we also discuss in lines 513-532, whether there is a relationship between mass-specific metabolic rate, cell division rate, and cancer is unclear. Human data exists for basal metabolic rate and cancer, from which one would in principle be able to derive allometric equations for the mass-specific rate and cancer, but this is strongly confounded by the multiple effects of obesity on physiology, and the extrapolation to non-human animals would be of doubtful utility.

Given that the relationship between longevity and flight is better documented, we simply assume birds gained flight which lowered their extrinsic mortality. Given that birds have lower cancer incidence than mammals, we argue that in our simulations, the parameter range that best corresponds to the observed bird life history involves miniaturisation with constant *k*. This is also the case where we found longer lifespans and lower cancer risk in birds than mammals — in line with the observed patterns. However, as e.g. potential bird-mammal differences in the number of steps until oncogenesis, *n*, remain undocumented, we prefer to show the effects for all scenarios instead of claiming that one small parameter region is the definite one.

4- I do not fully get the paradox about life span and cancer defence. Apparently Figure 4a shows both behaviors (p 10 "even as they continued to prolong life span (Fig 4a)" and "typically do not live long enough to enjoy...(Fig 4a)). This should be better explained (see later on data visualization). Trying to understand, I looked for the equations, and unless I am mistaken I could not find the explicit form of L(d) (only a reference to [54]). Also, in p7 L(d) is called 'reproductively active lifespan', and elsewhere simply as lifespan. This is an important point, since if I understand the two can be different (e.g., in introduction: longer life span, lower cancer risk but reduced reproductive success). Please, clarify.

What we mean with this is that in the lineages where the cost of cancer suppression is high, having a high level of cancer suppression can be maladaptive because it takes away from reproductive effort (the trade-off assumption); if reducing cancer suppression is the price one has to pay for higher reproductive output, we are here in the parameter region where this pays off. Shorter life with higher reproduction per unit time yields better fitness, in these precise situations, than a longer life with lower reproduction per unit time. This happens when the longer life is not *that* much longer, limited e.g. by extrinsic mortality that limits lifespan (in a stochastic manner) even if organisms were perfectly cancer-resistant. We now write about this more clearly (lines 533-542), and included the explicit form of L(d) in our manuscript (lines 245-247) and corrected the definition of L(d) to be the “expected lifespan” on page 8.

5 - I find the data visualization less than optimal. All data are in few Figures packed with density plots, and refereed generically in the main text as, eg, Figure 3 or Figure 3a, which contain lots of subplots. I had to go through them several times before getting the points. Hereafter, I give some criticisms and suggestions on how to improve them:

-p 10, different references to the same Figure 3C point to quite different things: "being constrained to express ancestral defences did not incur large fitness losses for the miniaturised lineages"; and "maintaining ancestral defences became maladaptive". This is confusing also because Figure 3C is made of four different density plots. In Figure 2 and 4 that are 6. Which of these plots are being referred to? Within the relevant density plots, I think it would help the reader if the three main quadrants were identified (low costs; high costs with yes/no substitutions; and so concerning my question, adding in the lower right corner 'maladaptive' and other labels in the other two regions).

- In figure 3 we have 4 density plots (two missing are in S6), in Figures 3 and 4 we have six of them. Why is that?

We opted to focus on four density plots in Figure 3, as lags, which are our main focus, are clearly shown in these. Given the constant-sized lineages start at and stay around their optimal level of cancer suppression, density plots remain simple in these cases, and we relegated these to the Supplementary Information. Having few plots in the main text and more complete sets in Supplementary Information is, in our view, a good compromise that allows the most interested readers to see everything, while being able to point out the most interesting cases in the main text.

- The most interesting comparisons are pairwise. If the Authors want to keep all density plots together, maybe they could organize them as a 2X2 table where pairwise comparisons are more easily detected

(something like

scaling k constant k

flight

flightless

).

I would then keep the two controls (ie, constant [small or large] constant k flightless) outside the table.

We would agree with this suggestion, were it not for the fact that there are always two response variables that we want to show: the substitutions (the genomic response) as well as the evolved defence (the phenotypic response). If we did 2 x 2 tables, we would presumably group the genomic responses together and the phenotypic ones likewise together. This would have the obvious downside of separating two outcomes from the same simulation; the other downside is that the locations of the (roughly) horizontally running thresholds are difficult to compare when some of them are on different rows. Thus, while we gave this some serious thought, on balance we decided not to implement this change.

Also, the meaning of the controls (eg, the mammal-like) may be stated explicitly in the methods section where they are introduced (p4).

Done (lines 133-135).

- Figure 2b-c, some of the low mutation rate trajectories are not easy to follow (do they go up and down?).

One of the main messages we wanted convey with Fig. 2b-c was that the low mutation rate trajectories behave differently from the high mutation rate ones (red), e.g. go down to the optimal level, or go down and then up to the optimal, or go down and stay suboptimal. To make sure the reader understands this indeed is the case, we discuss these observations in the section “Low mutation rate can result in phylogenetic inertia in cancer defences”.

- p 10 "all lineages evolved to their optimal level of cancer defences (Fig 2e)". Where do we see the optimal level in 2e?

We agree with the reviewer that this was unclear and have now added “as seen by the lack of fitness lags in (Fig. 3c)” to explain our reasoning (line 369).

- p 11 "kept the ancestrally high cancer defences useful Fig3", not clear to me where I see this in Fig 3.

As per above, we also added “(no fitness lags in Fig. 3c)” (line 424), since if they had fitness lags, that would imply that the ancestral cancer defences were maladaptive, rather than useful.

Minor comments:

- p3 "The life history option of a slow life history" needs to be rephrased

Done (line 78).

- p4

- "we contrast results" -> " we compare results"

Done (lines 131).

- end of introduction "lowering the effects of these evolutionary lags" should not it rather be 'diminuishing the evolutionary lags'?

Done (line 103).

- in the text on [46] -> in the text [46]

Done (line 140).

- "size and date data" -> not clear

Done (lines 142-143).

p6

- the concept of "rate of oncogenic steps" should be better introduced and given appropriate citations (eg, Vogelstein).

Done (lines 219-221).

- equation (1) is important, and derived in a previous paper. It should be briefly explained in this text to make it 'self-sustained'.

Done (lines 226-232).

- the fact that flightless, small sized from time 0 and constant k is a mammal-like lineage appears all of a sudden at p.9. The concept is relevant, and it should be introduced earlier (see main comment 5).

Done (lines 133-135, per above).

- If I am not mistaken, Figure 3b is never mentioned in the text. We find a description of its meaning in the legend. I think this part belongs to the text. In general, captions bear information that could be moved to the main text.

We now have shortened the captions of Figure 3 and Figure 4 and added the information to the main text (lines 335-338, 394-399, 407-409, 441-444).

- References to supplementary material should indicate precisely which figure in there they refer to.

Done.

- The first paragraph of Results is a new abstract, quite difficult to follow.

We agree and this paragraph is no longer in the MS.

- Legend in Figure 2a, flighted -> flight, to be consistent with all other figures

There is a subtle difference here: by “flighted” we mean that the lineage is flighted (a qualitative statement), while we use “flight” with information on timing (e.g. 170 Mya), i.e. a lineage switches from being flightless to flighted at that point in time.

Cited References

Abegglen LM, Caulin AF, Chan A, Lee K, Robinson R, Campbell MS, Kiso WK, Schmitt DL, Waddell PJ, Bhaskara S, Jensen ST, Maley CC,Schiffman JD. 2015. Potential mechanisms for cancer resistance in elephants and comparative cellular response to DNA damage in humans. JAMA 314:1850-1860.

Bergeron, L. A., Besenbacher, S., Zheng, J., Li, P., Bertelsen, M. F., Quintard, B., ... & Zhang, G. (2023). Evolution of the germline mutation rate across vertebrates. Nature, 615(7951), 285-291.

Dujon, A. M., Vincze, O., Lemaitre, J-F, Alix-Panabières, C., Pujol, P., Giraudeau, M., Ujvari, B., & Thomas, F. (2023). The effect of placentation type, litter size, lactation and gestation length on cancer risk in mammals. Proc. R. Soc. B, 290, 20230940.

Eyre-Walker, A., & Keightley, P. D. (2007). The distribution of fitness effects of new mutations. Nature Reviews Genetics, 8(8), 610-618.

Fernandez, A. A., & Bowser, P. B. (2010). Selection for a dominant oncogene and large male size as a risk factor for melanoma in the *Xiphophorus* animal model. Molecular Ecology, 19, 3114-3123.

Harper, J.M., Wang, M., Galecki, A.T., Ro, J., Williams, J.B. & Miller, R.A. 2011. Fibroblasts from long-lived bird species are resistant to multiple forms of stress. J. Exp. Biol. 214:1902-1910.

Kapsetaki, S.E., Compton, Z.T., Dolan, J., Harris, V.K., Mellon, W., Rupp, S.M., Duke, E.G., Harrison, T.M., Aksoy, S., Giraudeau, M., Vincze, O., McGraw, K.J., Aktipis, A., Tollis, M., Boddy, A.M. & Maley, C.C. 2024. Life history traits and cancer prevalence in birds. Evolution, Medicine, and Public Health 105-116.

Kapusta, A., Suh, A., & Feschotte, C. (2017). Dynamics of genome size evolution in birds and mammals. Proceedings of the National Academy of Sciences, 114(8), E1460-E1469.

Meitern, R., Fort, J., Giraudeau, M., Rattiste, K., Sild, E. & Sepp, T. (2020). Age-dependent expression of cancer-related genes in a long-lived seabird. Evol. Appl. 13:1708-1718.

Niebora, J., Data, K., Domagala, D., Jozkowiak, M., Barrett, S., Abbariki, T.N., Bryja, A., Kulus, M., Wozniak, S., Ziemak, H., Piotrowska-Kempisty, H., Antosik, P., Bukowska, D., Mozdziak, P., Dziegiel, P. & Kempisty, B. (2024). Avian models for human carcinogenesis— recent findings from molecular and clinical research. Cells 13:1797.

Nunney L. (2018). Size matters: height, cell number and a person’s risk of cancer. Proceedings of the Royal Society B: Biological Sciences, 285(1889). doi:10.1098/rspb.2018.1743.

Rietveld, L.E.G., Caldenhoven, E. & Stunnenberg, H.G. (2001). Avian erythroleukemia: a model for corepressor function in cancer. Oncogene 20:3100-3109.

Soriano-Redondo, A., Gutiérrez, J.S., Hodgson, D. & Bearhop, S. 2020. Migrant birds and mammals live faster than residents. Nature Comm. 11:5719.

Tollis, M., Robbins, J., Webb, A.E., Kuderna, L.F.K., Caulin, A.F., Garcia, J.D., Berube, M., Pourmand, N., Marques-Bonet, T., O'Connell, M.J., Palsboll, P.J. & Maley, C.C. (2019). Return to the sea, get huge, beat cancer: an analysis of cetacean genomes including an assembly for the humpback whale (Megaptera novaeangliae). Mol. Biol. Evol. 36:1746-1763.

Winger, B.M. & Pegan, T.M. 2021. Migration distance is a fundamental axis of the slow-fast continuum of life history in boreal birds. Ornithology 138:1-18.
