# Supplementary material for: Shrinking to bird size with dinosaur-level cancer defences: Evolution of cancer suppression over macroevolutionary time
Source: PLoS Comput Biol. 2025 Sep 9;21(9):e1013432. doi: 10.1371/journal.pcbi.1013432 (PMC12435733; doi:10.1371/journal.pcbi.1013432)
Supplement: Attachment — Submitted filename: Response to reviewers.docx [file pcbi.1013432.s004.docx]

25 06 2025

Dear Editor,

We hereby submit a revised version of our manuscript PCOMPBIOL-D-24-01081R1 “Shrinking to bird size with dinosaur-level cancer defences: evolution of cancer suppression over macroevolutionary time” for review in *PLOS Computational Biology*.

Please find below our response to the reviewer comments.

We hope you find our paper suitable for publication in *PLOS Computational Biology*.

Best regards (on behalf of the authors),

Dr. E. Yagmur Erten

Groningen Institute for Evolutionary Life Sciences (GELIFES)

University of Groningen

The Netherlands

Response to Reviewer Comments

Below we address the reviewer comments and describe how we revised our manuscript. Reviewer comments are shown in black and our responses are in blue.

**Reviewer #1:**

I appreciate the authors' detailed and thoughtful responses to my comments & concerns. They have enhanced and clarified my understanding of the design and intent of their work.

We thank the reviewer for these nice statements and also for their critical comments below.

However, I would have liked the information in their responses to be incorporated directly into the revised manuscript in some way, beyond the addition at lines 606-609 regarding potential pathways for future use of their model to inform genomic and phenotypic data. I feel this would improve the ability of first-time readers to understand and appreciate the intent and approach of this study, and thus be more likely incorporate the authors' frameworks and ideas into their own research.

We have extended the last paragraph of Discussion to incorporate this (lines 537-544).

The 2024 paper by Kapsetaki et al actually does provide some validation of the sort I proposed. It was not mentioned in their response to my comments but I see was referred to in response to other reviewers and is referenced in the manuscript. The association between brood size and cancer incidence across birds is encouraging. But Kapsetaki did not report any association of cancer rates with body size, which was one of the key elements of the authors' model. I'd therefore be interested in the authors' perspective on how that negative findings aligns with their models predictions, which I feel was further enrich this paper.

Our modelling approach is based on the idea that Peto's paradox (no association between cancer prevalence and body size across species) can be explained by selection for stronger cancer suppression in larger species (e.g. elephants or dinosaurs) than smaller ones (e.g. birds). In Kapsetaki et al. 2024, the authors show no association between body size and cancer prevalence in birds, in line with the previous papers on Peto’s paradox (except Bulls 2022, Compton et al. 2025, and Butler et al. 2025). This supports our modelling approach for the bird lineage. We now mention the results of Kapsetaki et al. 2024 in the Introduction (lines 49-51).

**Reviewer #3:**

The revised version of the manuscript in my opinion is only relatively improved. As before, I find the paper interesting, but it is hard to read and to follow. All the suggestions given for improving the figures have not been accepted, which can be fine of course, provided the Authors had tried alternative ways to improve the paper. This, it seems to me, was not done.

We thank the reviewer for their suggestions for improvement.

1- I understand that the model is complex, it includes many features and it is hard to explain.

Exactly for this reason it would make sense to introduce the reader to the questions more

clearly. The first paragraph of the results 'Reduced optimal cancer suppression in shrinking lineages' is very hard to follow. There is no introduction about the question addressed. Figure 2a requires a knowledge about almost everything: substitutions, lifespan, metabolic scaling, flight...to further complicate things, the reader is taken to Figure 4b before any other Figure (the generic reference Figures 2-4 cover the whole paper). I find it much easier to start the paper from the second paragraph. Many of the concepts introduced in the first paragraph are introduced later again anyway. I am not suggesting to remove the first paragraph, but I invite the authors to be more pedantic in what they ask and what they find, assuming that some readers may well decide to start reading from the Results section. A starting point is to make sure the Figures are cited in the correct order in the text.

We have now made changes throughout the Results section to increase the readability:

- We have rearranged and edited the first three sections of the Results for increased clarity.
- We have added the question we are studying to the beginning of the Results section (lines 309-312).
- We have changed the figures in line with the suggestions from the Reviewer 2 (see below).
- We now refer to the figures in correct order, and also more specifically by panel label than generically (e.g. Fig 2d, instead of Fig 2).
- Throughout the Results section, we have also added additional explanations to help with the interpretation of the figures (e.g. line 367).

2 - I still think that it would be very useful to have the figures in panels 2X2

scaling constant

flightless panel panel

flight panel panel

so that on the x on the scaling changes and on the y only the ability to fly. For example,

it would emerge very clearly that flight has the largest effect on lifespan. The two additional

panels with large vs small can be added in the same figure.

We thank the reviewer for this suggestion and have implemented it in this revision, streamlining Figs 2, 3, and 4 from the previous version into Figs 2 and 3, with the suggested panel structure and moving the results from the constant-sized lineages to the Supplementary Information (Fig. S6 and S7).

Other comments:

1 - In the text again and again it is reported that increased metabolic rate goes together

with increased risk of cancer (from the abstract onward: "an increase in cancer risk due to metabolic scaling"). Then we find in the Discussion and in the rebuttal that the model holds also when this is not the case "our results remain qualitatively similar whether metabolic 485 rate is assumed to correlate with cancer risk or not". I find this confusing. The authors should clarify this point.

Lower mass-specific metabolic rate in larger animals has been suggested as a factor that can explain Peto paradox, if rate of oncogenesis is correlated with mass-specific metabolic rate. However, the link between the rate of oncogenesis and mass-specific metabolic rate is unclear. Nevertheless, we included this allometric scaling aspect as one of our model variants to explore the possibility, for completeness. In the Discussion, we have edited the paragraph on metabolic rate with the aim of clarifying why we modelled metabolic scaling and its potential effect on cancer risk (lines 477-491).

2- the authors write about 'realistic' mutation rates (line. 95) and about 'more realistic scenario' (line 355), but in their rebuttal they argue, if I understand from their answers, that giving realistic ranges is not possible.

Here, we refer to the “realistic” mutation rates, as in comparable to what is estimated for birds, per genome per generation, but the “real” mutation rates would indeed, as we argued in our previous rebuttal, depend on various factors.

3- line 320: what does 'importance' mean? Where do I see this?

We have rephrased this: “We assumed that this lineage underwent a gradual decrease in body size, which shifted two sources of death in opposite directions: extrinsic mortality increased, whereas cancer risk decreased, compared to the birds' dinosaurian ancestors.” (line 312-314).

4- I am not sure what the Authors mean for pleiotropy, it is used to explain the mutation limited regime, but also in alternative to it (line 87).

Here, we mean that mutation rate being low could result from overall low mutation rate, as well as pleiotropic effects limiting mutation rate of specific genes. We have added “and/or” between the two (instead of “and”) to reflect this (line 89).

5- line 309: cancer risk decreased -> compared to?

As also mentioned in point 3 above, we have rephrased this: “We assumed that this lineage underwent a gradual decrease in body size, which shifted two sources of death in opposite directions: extrinsic mortality increased, whereas cancer risk decreased, compared to the birds' dinosaurian ancestors.” (line 312-314).

6- Figure 2b and 2c are not changed, and remain hard to understand to me. I do not get the blu

trajectories: do they go up and down? Only down? Is it possible to find a representation that shows

clearly the dynamics of each trajectory?

We thank the reviewer for highlighting this. We have changed Figure 2 to show some examples of the individual trajectories (Figure 2e-j), while keeping the Figure 2d (Figure 2b-c in the previous version) as is to show an overview.

7- The last raw of Figure 4 was not introduced in the text, unless I am mistaked.

This figure is no longer in the text, as described above.

8- I find the Discussion very long, when compared to the Results section.

In this version, we have reorganised the Discussion and shortened some parts of it (e.g. lines 477-491), while adding the changes suggested by Reviewer 1.

**References**

Bulls SE, Platner L, Ayub W, Moreno N, Arditi JP, Dreyer S, et al.. Unraveling the relationship between cancer and life history traits in vertebrates; 2024. Available from:

https://www.biorxiv.org/content/early/2024/07/24/2022.07.12.499088.

Butler G, Baker J, Amend SR, Pienta KJ, Venditti C. No evidence for Peto’s paradox in terrestrial vertebrates. Proceedings of the National Academy of Sciences. 2025;122(9):e2422861122. doi:10.1073/pnas.2422861122.

Compton ZT, Mellon W, Harris VK, Rupp S, Mallo D, Kapsetaki SE, et al. Cancer prevalence across vertebrates. Cancer discovery. 2025;15(1):227–244.

Kapsetaki SE, Compton ZT, Dolan J, Harris VK, Mellon W, Rupp SM, et al. Life history traits and cancer prevalence in birds. Evolution, Medicine, and Public Health. 2024;12(1):105–116.
